# Identification of key metabolic indicators associated with the comorbidity of ischemic stroke and diabetes mellitus using an optimal interpretable clinlabomics model

**DOI:** 10.3389/fcvm.2026.1874711

**Published:** 2026-06-24

**Authors:** Yao Jiang, Ao Qian, Shu Chen, Qian Wu, Hao Xu, Chang Zheng, Fengyu Zhang, Wenli Xing, Jimin He

**Affiliations:** 1 Department of Clinical Laboratory Medicine, Suining Central Hospital, Suining, China; 2 Department of Cerebrovascular Disease, Suining Central Hospital, Suining, China; 3 Faculty of Medical Technology, Shaanxi University of Chinese Medicine, Xi'an, China; 4 Department of Neurosurgery, Suining Central Hospital, Suining, China

**Keywords:** clinlabomics model, comorbidity, diabetes mellitus, ischemic stroke, machine learning, metabolic indicators

## Abstract

**Background:**

To identify candidate metabolic biomarkers and establish a Clinlabomics model for early precise screening of ischemic stroke (IS) with diabetes mellitus (DM) comorbidity population.

**Methods:**

A total of 2,587 IS patients were retrospectively enrolled and classified into IS-DM comorbidity and IS-only groups. A total of 16 metabolic indicators were collected, and candidate indicators were identified using univariate and multivariate logistic regression along with restricted cubic spline (RCS) analysis. The dataset was randomly split into training and test sets at a 7:3 ratio, and an additional 406 patients constituted a temporal validation set. Clinlabomics models were constructed using 11 machine learning (ML) algorithms. Model performance was evaluated using F1-score, accuracy (ACC) and area under the curve (AUC) to select the optimal algorithm, and SHapley Additive exPlanations (SHAP) analysis was performed to quantify feature contributions.

**Results:**

Multivariate logistic regression showed that 12 metabolic indicators were closely associated with IS-DM comorbidity. Notably, the triglyceride glucose (TyG) index (OR = 4.76, 95% CI: 4.01–5.64, *P* < 0.001) and atherogenic index of plasma (AIP) (OR = 3.60, 95% CI: 2.74–4.72, *P* < 0.001) were significantly associated with an increased risk of IS-DM comorbidity. RCS revealed non-linear associations between 9 indicators and comorbidity. Least Absolute Shrinkage and Selection Operator (LASSO) analysis identified 16 feature variables, and the recursive partitioning and regression trees (rpart) algorithm model achieved the best performance, with ACC, F1-score, AUC, sensitivity, and specificity of 0.885, 0.818, 0.910, 78%, and 94%, respectively. The AUC values in the training, test and temporal validation sets were 0.910 (95% CI: 0.894–0.926), 0.839 (95% CI: 0.807–0.871) and 0.873 (95% CI: 0.831–0.915), respectively. SHAP analysis suggested that glycated hemoglobin A1c (HbA1c)/high-density lipoprotein cholesterol (HDL-C) was the most important feature. Finally, 9 candidate metabolic biomarkers for comorbidity were identified, including TyG, uric acid (UA), total cholesterol (TC), low-density lipoprotein cholesterol (LDL-C), AIP, atherogenic coefficient (AC), Castelli's risk index I (CRI-I), Castelli's risk index II (CRI-II), and HbA1c/HDL-C.

**Conclusions:**

This study successfully identified 9 candidate metabolic indicators of IS-DM comorbidity. The Clinlabomics model established by rpart algorithm presents excellent performance in identifying the IS-DM population.

## GRAPHICAL ABSTRACT

**Figure:**
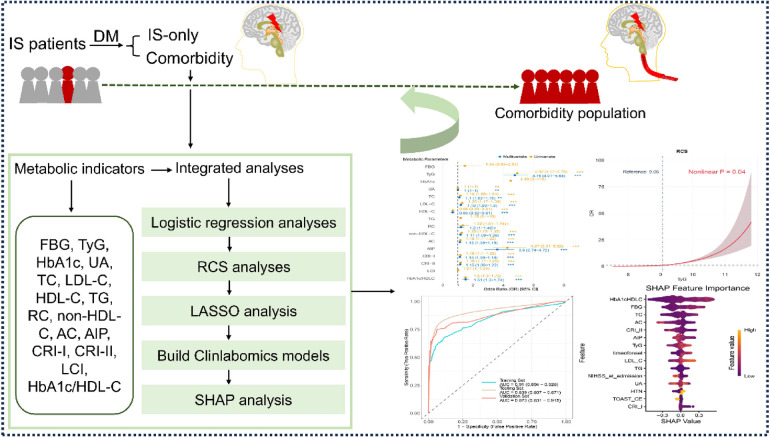

## Introduction

Ischemic stroke (IS) remains a leading cause of long-term disability and mortality worldwide, imposing a heavy socioeconomic burden on global public health systems (1). Globally, IS constitutes 65.3% (95% CI: 62.4–67.7) of all new-onset stroke cases (1). In recent years, the disease burden of IS has been continuously rising. Studies indicate that IS-related deaths will further increase from 3.29 million in 2019 to 4.90 million in 2030, posing an increasingly grave challenge to public health prevention and control (2). Diabetes mellitus (DM), as one of the risk factors for IS, not only increases stroke susceptibility but also impairs neurovascular repair through persistent systemic inflammation, endothelial dysfunction, and impaired neuroplasticity (3, 4). Epidemiological data demonstrate the high prevalence of comorbid IS and DM. Specifically, a meta-analysis showed that up to 33% (95% CI: 28%–38%) of patients with IS worldwide have DM (5). National Chinese registry data also confirm an upward trend in the prevalence of type 2 diabetes mellitus (T2DM) among IS patients, rising from 12.0% during 2004–2006 to 17.0% in 2019–2020. Moreover, 30-day and 1-year readmission rates were consistently higher in IS patients with comorbid T2DM (6). Prospective data analysis from the China National Stroke Registry 3 (CNSR-3) further demonstrates that patients with acute ischemic stroke (AIS) in China bear a substantial comorbidity burden. Among these comorbidities, DM ranks as one of the most prevalent (third overall), accounting for 31.38% (7). After stroke, DM patients exhibit approximately 25% lower likelihood of achieving favorable prognosis and higher stroke-related mortality risk; compared with nondiabetic individuals, diabetic stroke survivors have elevated risks of recurrent stroke and subsequent vascular cognitive impairment (8). This figure clearly underscores the clinical severity of the comorbid condition of IS and DM, as well as the urgent need for its prevention and control.

In clinical practice, standardized management for patients with concomitant IS and DM relies heavily on accurate risk stratification and individualized interventions. Identifying biomarkers that objectively reflect the pathophysiology underlying this comorbidity represents a core step toward this goal. Analysis of the CNSR-3 dataset underscores that hypertension (HTN), dyslipidemia, and DM, as three of the most prevalent comorbidities in AIS, are major metabolic conditions closely associated with stroke pathogenesis (7). Concurrent DM and dyslipidemia are markedly associated with increased stroke risk (OR = 3.64) (9), while machine learning models incorporating inflammatory and metabolic biomarkers (e.g., glycated hemoglobin A1c [HbA1c], triglyceride-glucose [TyG] index) effectively predict 1-year unplanned readmission in patients with concomitantIS and DM, consistent with metabolic dysregulation being closely linked to adverse outcomes (10). Systemic metabolic disturbance represents a central pathological correlate of IS-DM comorbidity, rendering metabolic parameters the most direct and clinically valuable biomarkers for characterizing this condition. Routine clinical laboratory indicators are convenient, reproducible, and widely available, allowing comprehensive evaluation of systemic metabolic status. However, the key metabolic signatures and underlying molecular mechanisms of these indicators in patients with concomitant IS and DM remain poorly and systematically elucidated. Wang et al. proposed the concept of Clinlabomics, which integrates mid-dimensional clinical laboratory parameters with machine learning (ML) algorithms to provide a novel strategy for disease early warning and prognostic evaluation (11). In recent years, researchers have developed Clinlabomics models to predict therapeutic response to immune checkpoint inhibitors (ICIs) in patients with advanced gastric cancer (12), screen for primary angle-closure glaucoma (13), identify AIS phenotypes (14), and evaluate prognosis among Chinese patients with small cell lung cancer (15). Furthermore, our team previously constructed an optimal Clinlabomics model for patients with concomitant IS and hyperuricemia (HUA) and determined its key metabolic parameters (16), laying a solid methodological and practical foundation for the present study.

This study aimed to include 16 routine and derived clinical laboratory metabolic indicatorsand explore their associations with concomitant IS-DM comorbidity. We used restricted cubic spline (RCS) analysis to explore potential nonlinear relationships, constructed Clinlabomics models via 11 ML algorithms, and employed the SHapley Additive exPlanations (SHAP) approach to interpret the black-box nature of the optimal model and quantify the contribution of each indicator. This transparent, data-driven framework was designed to enable rapid identification of individuals with concomitant IS and DM.

## Materials and methods

### Study population

**Figure 1:**
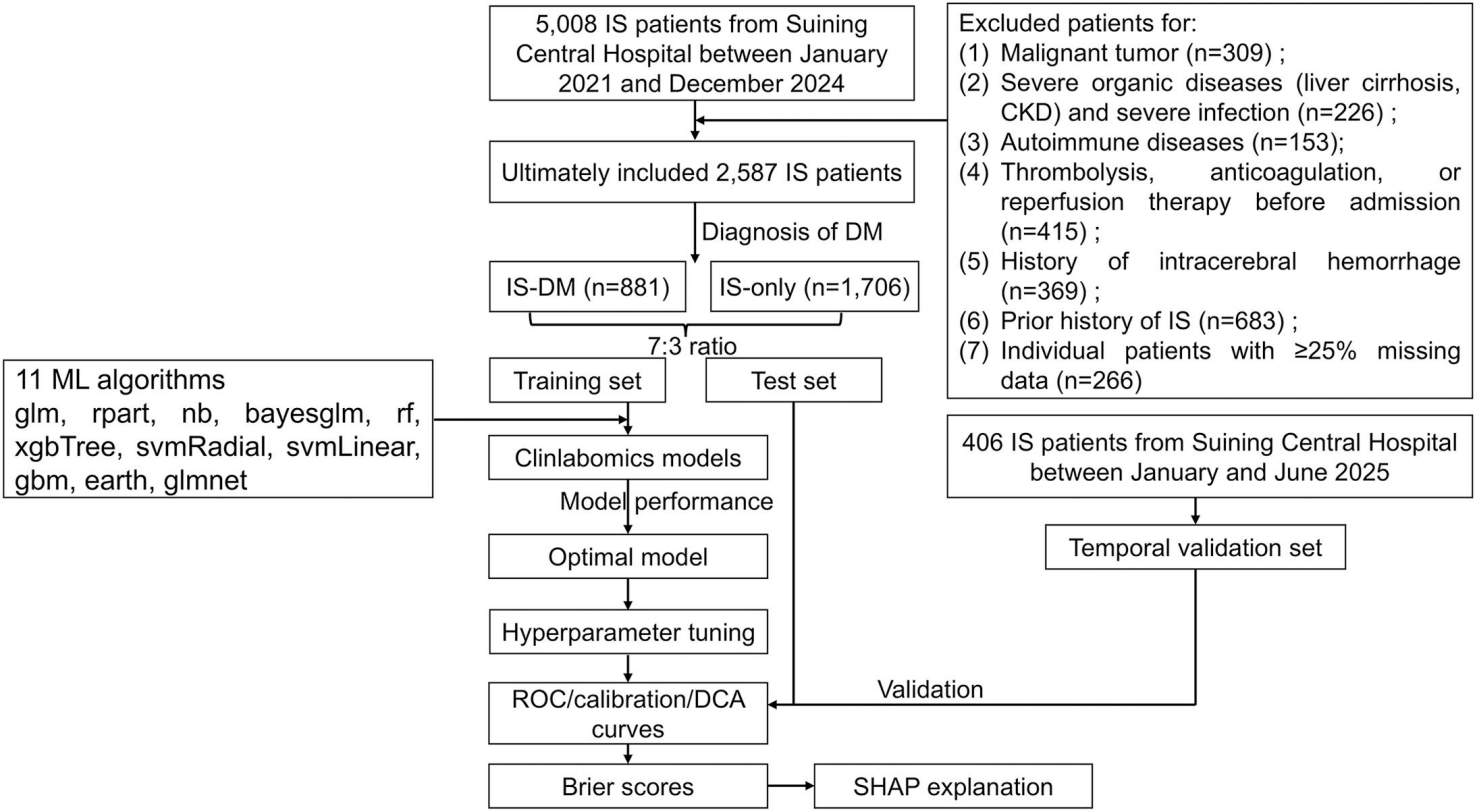
Flowchart of the present study.

We recruited a total of 5,008 patients with IS from January 2021 to December 2024. The inclusion criteria for IS was first-onset stroke at admission, age ≥18 years, and definite diagnosis confirmed by computed tomography (CT) and/or magnetic resonance imaging (MRI) on admission. Patients were excluded if they had malignancy, severe organic diseases (hepatic insufficiency, liver cirrhosis, chronic kidney disease [CKD]), severe infection, autoimmune diseases, received anticoagulant, thrombolytic or reperfusion therapy prior to admission, presented with intracerebral hemorrhage, were pregnant or lactating women, had a previous history of IS, or had a missing data rate ≥25%. Ultimately, 2,587 IS patients were included in the study, and an additional 406 eligible IS patients from January to June 2025 were enrolled as the temporal validation cohort. This study was conducted in accordance with the ethical principles of the Declaration of Helsinki and approved by the Ethics Committee of Suining Central Hospital (No. KYLLKS20250126). All participants provided written informed consent prior to enrollment. A detailed flowchart illustrating all analytical procedures of the present study is presented in Figure 1.

### Data collection

Clinical data at admission were extracted from electronic medical records (EMRs), including gender, age, medical history (hypertension [HTN], DM, coronary artery disease [CHD], atrial fibrillation [AF]), smoking and alcohol consumption history, medication history (antiplatelet agents, statins), Trial of Org 10,172 in Acute Stroke Treatment (TOAST) classification, time from symptom onset to admission, baseline National Institutes of Health Stroke Scale (NIHSS) score (17), and baseline systolic/diastolic blood pressure (SBP/DBP), NIHSS score at discharge, and modified Rankin Scale (mRS) score (18) at discharge. DM was defined as a preadmission diagnosis treated with hypoglycemic agents or insulin. New-onset DM after admission was diagnosed according to standard criteria (19): HbA1c ≥6.5%, fasting blood glucose (FBG) >7.0 mmol/L, 2 h plasma glucose ≥11.1 mmol/L during a 75 g oral glucose tolerance test (OGTT), or random blood glucose ≥11.1 mmol/L accompanied by typical hyperglycemic symptoms. Laboratory parameters measured at admission were collected via the Laboratory Information System (LIS), including white blood cell count (WBC), neutrophil count (NEU), lymphocyte count (LYM), monocyte count (MON), platelet (PLT), FBG, glycated hemoglobin A1c (HbA1c), uric acid (UA), total cholesterol (TC), low-density lipoprotein cholesterol (LDL-C), high-density lipoprotein cholesterol (HDL-C), and triglyceride (TG).

### Metabolic parameters measurement

FBG was determined by the hexokinase method, while UA was measured with the uricase-peroxidase technique. TC and TG were quantified via the cholesterol oxidase method and glycerol-3-phosphate oxidase-peroxidase (GPO-POD) method, respectively. HDL-C and LDL-C were examined using direct assays. These metabolic parameters were measured using the Beckman Coulter AU5800 system. HbA1c was detected by affinity high-performance liquid chromatography (HPLC) via affinity HPLC HbA1c analyzer (Premier Hb9210). The formulas for calculating the derived inflammatory indicators are as follows: systemic inflammation index (SII) = PLT * NEU/LYM (20), system inflammation response index (SIRI) = NEU*MON/LYM (20). The derived metabolic indicators are listed below: triglyceride-glucose index (TyG) = ln(FBG (mg/dL) * TG (mg/dL)/2) (21), remnant cholesterol (RC) = TC—HDL-C—LDL-C (22), atherogenic index of plasma (AIP) = lg(TG/HDL-C) (23), non-HDL-C = TC—HDL-C, atherogenic coefficient (AC) = non-HDL-C/HDL-C (24), lipoprotein combine index (LCI) = TC *TG * LDL-C/HDL-C (25, 26), Castelli's risk index I (CRI-I) = TC/HDL-C (24), and Castelli's risk index II (CRI-II) = LDL-C/HDL-C (24), and the ratio of HbA1c/HDL-C (27).

### Data processing and dataset partitioning

First, outliers were identified via the interquartile range (IQR) method and processed robustly. Besides, data with a missing rate of <25% need processed, but direct imputation of the entire dataset must be avoided to prevent information leakage. Therefore, we used the “createDataPartition” function in the “caret” package to randomly split the dataset into a training set and a test set at a ratio of 7:3. Multiple imputation was then performed only on the training set using the “mice” package. Subsequently, the imputation model derived from the training set was directly applied to the test set and the temporal validation set, and no separate imputation was performed on these two datasets.

### Feature variable selection

In the training set, LASSO analysis was conducted on differentially expressed clinical features and metabolic indicators to identify key variables. Notably, continuous variables were standardized during the LASSO feature-selection stage.

### Development of clinlabomics models

Eleven machine learning (ML) algorithms, including generalized linear model (glm), recursive partitioning and regression trees (rpart), naïve bayes (nb), bayesian generalized linear model (bayesglm), lasso and elastic-net regularized generalized linear models (glmnet), multivariate adaptive regression splines (earth), gradient boosting machine (gbm), random forest (rf), support vector machine with linear kernel (svmLinear), support vector machine with radial basis function kernel (svmRadial), extreme gradient boosting tree (xgbTree), were applied to construct comorbidity models in the training set; detailed information on these ML algorithms can be found in our previously published study (16). Model performance was evaluated using accuracy (ACC), F1-score, sensitivity (SEN), specificity (SPE), and area under the curve (AUC) to identify the optimal Clinlabomics model with superior performance in the training set. Hyperparameter tuning for the optimal model was conducted in the training set via grid search and 5-fold cross-validation to identify the best hyperparameter settings. Receiver operating characteristic (ROC) curves, calibration curves, and decision curve analysis (DCA) were generated for the optimal model in the three datasets, respectively. Additionally, the Brier score was used to comprehensively assess the discrimination and calibration performance of the model across the three datasets (ranging from 0 to 1), with lower values indicating that the probabilities estimated by the model were closer to the actual outcomes (28). Furthermore, to interpret the contribution of individual features to the model, the SHAP algorithm was used to calculate SHAP values from the best-performing Clinlabomics model. SHAP values quantify the marginal contribution of each feature to the predicted outcome, and this method supports both local and global interpretability of the model. The entire development process of the Clinlabomics model was implemented using the “Icare” package (https://github.com/OmicsLY/Icare).

### Statistical analysis

Normality testing for numerical data was conducted using the Kolmogorov-Smirnov test. Normally distributed data were presented as mean ± standard deviation, and between-group differences were compared using the Student's *t*-test. Non-normally distributed continuous variables were described as median (Q1, Q3), and between-group differences were analyzed using the Mann–Whitney *U*-test. Subsequently, univariate and multivariate logistic regression analyses were performed to explore the associations between metabolic indicators and IS-DM comorbidity. The adjusted covariates included gender, age, time from symptom onset to admission, HTN, CHD, AF, antiplatelet therapy, statin therapy, TOAST classification, smoking, drinking, SBP, DBP, admission NIHSS score, WBC, and SII. As TOAST classification is an unordered multinomial variable, dummy variable coding was applied before regression. Using the large-artery atherosclerosis (LAA) subtype as the reference, binary dummy variables were constructed for cardioembolism (CE), small-artery occlusion (SAO), stroke of other etiology (SOE), and stroke of undetermined etiology (SUE), respectively, with a uniform 0/1 coding scheme. Only these dummy variables were included in the subsequent multivariate regression model to eliminate multicollinearity. Subsequently, restricted cubic spline (RCS) analysis was performed to explore the non-linear relationships between metabolomic indicators and IS-DM comorbidity, with four knots set at the 5th, 35th, 65th, and 95th percentiles. Additionally, we performed sensitivity analyses by removing HbA1c, FBG and the FBG-based TyG index to rule out circular reasoning. All statistical analyses were performed with RStudio (Version 4.3.0). A two-tailed *P* < 0.05 was considered statistically significant.

## Results

### Basic characteristics of included IS population

Missing laboratory data with a missing rate <25% were processed by multiple imputation, including WBC (1.9%), NEU (1.9%), LYM (1.9%), MON (1.9%), PLT (1.9%), FBG (6.1%), HbA1c (11.6%), UA (1.3%), TC (7.0%), LDL-C (7.0%), HDL-C (7.0%), TG (7.0%). Supplementary Table S1 presents the number and proportion of missing values and distribution comparisons before and after imputation, with no significant differences detected (all *P* > 0.05). This cohort comprised elderly patients with a median age of 72 years, and males accounted for 58%. The median time of IS onset was 6 h. Median NIHSS scores were 5 (2, 9) at admission and 3 (1, 8) at discharge, with a median discharge mRS score of 2 (1, 4). Between-group comparisons showed significant differences in age, gender, time of onset, TOAST classification, NIHSS at admission, HTN, AF, SBP, WBC, NEU, LYM, SIRI, FBG, TyG, HbA1c, UA, TC, LDL-C, HDL-C, TG, RC, non-HDL-C, AC, AIP, LCI, CRI-Ⅰ, CRI-Ⅱ and HbA1c/HDL-C between the IS-DM comorbidity and IS-only groups (Table 1, *P* < 0.05). After dataset splitting at a 7:3 ratio, these metabolic indicators remained significantly different in the training set (Table 2, *P* < 0.05). Supplementary Table S2 presents detailed test set results. In the temporal validation cohort, levels of FBG, TyG, HbA1c, TC, LDL-C, TG, non-HDL-C, AC, AIP, LCI, CRI-Ⅰ, CRI-Ⅱ and HbA1c/HDL-C were also significantly elevated in the IS-DM comorbidity group (Table 3, *P* < 0.05). The between-group trends of metabolic indicators were consistent across training and validation sets, with stable data distribution, which ensures the reliability of the established models.

**Table 1:** The main characteristics of included IS patients.

Variables	Total (*n* = 2,587)	IS-only (*n* = 1,706)	IS-DM (*n* = 881)	*P*-value
Age (years)	72 (64, 79)	72 (64, 79)	71 (63, 78)	**0.003**
Gender, *n* (%)				**0.014**
Female	1,093 (42)	691 (41)	402 (46)	
Male	1,494 (58)	1,015 (59)	479 (54)	
Time of onset (h)	6 (2, 24)	5 (2, 24)	6 (2, 24)	**0.030**
TOAST, *n* (%)				**<0.001**
CE	478 (19)	376 (22)	102 (12)	
LAA	785 (30)	508 (30)	277 (31)	
SAO	1,220 (47)	745 (44)	475 (54)	
SOE	55 (2)	44 (2)	11 (1)	
SUE	49 (2)	33 (2)	16 (2)	
HTN, *n* (%)				**<0.001**
No	1,018 (39)	749 (44)	269 (31)	
Yes	1,569 (61)	957 (56)	612 (69)	
CHD, *n* (%)				0.187
No	2,365 (91)	1,569 (92)	796 (90)	
Yes	222 (9)	137 (8)	85 (10)	
AF, *n* (%)				**<0.001**
No	2,144 (83)	1,356 (79)	788 (89)	
Yes	443 (17)	350 (21)	93 (11)	
Antiplatelet therapy, *n* (%)				0.882
No	2,558 (99)	1,686 (99)	872 (99)	
Yes	29 (1)	20 (1)	9 (1)	
Statin therapy, *n* (%)				1
No	2,561 (99)	1,689 (99)	872 (99)	
Yes	26 (1)	17 (1)	9 (1)	
Smoking, *n* (%)				0.185
No	1,910 (74)	1,245 (73)	665 (75)	
Yes	677 (26)	461 (27)	216 (25)	
Drinking, *n* (%)				0.622
No	2,111 (82)	1,387 (81)	724 (82)	
Yes	476 (18)	319 (19)	157 (18)	
SBP	145 (128, 163)	145 (128, 162)	148 (130, 164)	**0.017**
DBP	84 (74, 94)	84 (74, 95)	84 (73, 94)	0.260
NIHSS at admission	5 (2, 9)	5 (2, 10)	4 (2, 8)	**<0.001**
NIHSS at discharge	3 (1, 8)	3 (1, 8)	3 (1, 7)	0.625
mRS at discharge	2 (1,4)	2 (1,4)	2 (1,4)	0.701
WBC (×10^9^)	7.5 (6.0, 9.5)	7.4 (5.9, 9.5)	7.7 (6.2, 9.6)	**0.003**
NEU (×10^9^)	5.25 (3.94, 7.28)	5.16 (3.88, 7.30)	5.40 (4.10, 7.24)	**0.040**
LYM (×10^9^)	1.34 (0.94, 1.83)	1.30 (0.92, 1.80)	1.44 (1.04, 1.92)	**<0.001**
MON (×10^9^)	0.47 (0.36, 0.62)	0.46 (0.36, 0.62)	0.47 (0.36, 0.63)	0.703
PLT (×10^9^)	187 (142, 232)	184 (142, 231)	190 (142, 234)	0.361
SII	700 (425, 1,247)	720 (426, 1,271)	659 (420, 1,198)	0.113
SIRI	1.73 (1.05, 3.30)	1.79 (1.09, 3.42)	1.65 (1.02, 3.18)	**0.045**
FBG (mmol/L)	7.40 (6.04, 10.30)	6.60 (5.66, 8.00)	11.36 (8.20, 15.46)	**<0.001**
TyG	9.06 (8.58, 9.66)	8.82 (8.42, 9.27)	9.67 (9.17, 10.21)	**<0.001**
HbA1c (%)	6.2 (5.8, 6.9)	5.9 (5.6, 6.2)	7.7 (6.5, 9.6)	**<0.001**
UA (*μ*mol/L)	333.0 (270.0, 407.0)	340.0 (275.0, 415.0)	321.0 (262.0, 396.0)	**<0.001**
TC (mmol/L)	4.91 (4.10, 5.71)	4.83 (4.03, 5.59)	5.08 (4.25, 5.92)	**<0.001**
LDL-C (mmol/L)	2.91 (2.31, 3.53)	2.82 (2.26, 3.45)	3.07 (2.42, 3.74)	**<0.001**
HDL-C (mmol/L)	1.30 (1.09, 1.56)	1.33 (1.12, 1.60)	1.26 (1.04, 1.50)	**<0.001**
TG (mmol/L)	1.38 (0.97, 2.06)	1.24 (0.89, 1.83)	1.69 (1.17, 2.61)	**<0.001**
RC (mmol/L)	0.54 (0.32, 0.87)	0.52 (0.30, 0.82)	0.58 (0.35, 0.97)	**<0.001**
non-HDL-C (mmol/L)	3.52 (2.78, 4.33)	3.37 (2.72, 4.16)	3.76 (2.96, 4.60)	**<0.001**
AC	2.68 (1.99, 3.47)	2.54 (1.90, 3.31)	2.93 (2.27, 3.82)	**<0.001**
AIP	0.03 (−0.16, 0.24)	−0.02 (−0.21, 0.18)	0.13 (−0.06, 0.35)	**<0.001**
LCI	14.94 (7.49, 30.22)	12.47 (6.64, 24.48)	20.76 (10.86, 42.32)	**<0.001**
CRI-I	3.68 (2.99, 4.47)	3.54 (2.90, 4.31)	3.93 (3.27, 4.82)	**<0.001**
CRI-II	2.24 (1.69, 2.85)	2.11 (1.62, 2.73)	2.44 (1.87, 3.07)	**<0.001**
HbA1c/HDL-C	4.92 (3.92, 6.29)	4.43 (3.65, 5.39)	6.31 (4.93, 8.30)	**<0.001**

IS, ischemic stroke; TOAST, Trial of Org 10,172 in Acute Stroke Treatment; CE, Cardioembolism; LAA, large artery atherosclerosis; SAO, small artery occlusion; SOE, stroke of other etiology; SUE, stroke of undetermined etiology; HTN, hypertension; CHD, coronary heart disease; AF, atrial fibrillation; SBP, systolic blood pressure; DBP, diastolic blood pressure; NIHSS, National Institutes of Health Stroke Scale; mRS, modified Rankin Scale; WBC, white blood cell; NEU, neutrophil; LYM, lymphocyte; MON, monocyte; PLT, platelet; SII, systemic inflammation index; SIRI, system inflammation response index; FBG, fasting blood glucose; TyG, triglyceride-glucose index; HbA1c, glycated hemoglobin A1c; UA, uric acid; TC, total cholesterol; LDL-C, low density lipoprotein cholesterol; HDL-C, high density lipoprotein cholesterol; TG, triglyceride; RC, remnant cholesterol; AC, atherogenic coefficient; AIP, atherogenic index of plasma; LCI, lipoprotein combine index; CRI-I, Castelli's risk index I; CRI-II, Castelli's risk index II; bold font indicates statistically significant differences.

**Table 2:** The characteristics of IS patients in the training set.

Variables	IS-only (*n* = 1,206)	IS-DM (*n* = 605)	*P*-value
Age (years)	72 (64, 79)	71 (63, 77)	**0.013**
Gender, *n* (%)			0.160
Female	493 (41)	269 (44)	
Male	713 (59)	336 (56)	
Time of onset (h)	5 (2, 24)	7 (2, 24)	**0.009**
TOAST, *n* (%)			**<0.001**
CE	263 (22)	71 (12)	
LAA	361 (30)	202 (33)	
SAO	527 (44)	315 (52)	
SOE	28 (2)	7 (1)	
SUE	27 (2)	10 (2)	
HTN, *n* (%)			**<0.001**
No	521 (43)	176 (29)	
Yes	685 (57)	429 (71)	
CHD, *n* (%)			0.137
No	1,111 (92)	544 (90)	
Yes	95 (8)	61 (10)	
AF, *n* (%)			**<0.001**
No	961 (80)	537 (89)	
Yes	245 (20)	68 (11)	
Antiplatelet therapy, *n* (%)			1
No	1,195 (99)	600 (99)	
Yes	11 (1)	5 (1)	
Statin therapy, *n* (%)			1
No	1,197 (99)	600 (99)	
Yes	9 (1)	5 (1)	
Smoking, *n* (%)			0.528
No	895 (74)	458 (76)	
Yes	311 (26)	147 (24)	
Drinking, *n* (%)			0.788
No	987 (82)	499 (82)	
Yes	219 (18)	106 (18)	
SBP	144 (127, 162)	147 (130, 164)	0.057
DBP	84 (74, 94)	83 (72, 93)	0.242
NIHSS at admission	5 (2, 10)	4 (2, 8)	**0.005**
NIHSS at discharge	4 (1, 8)	3 (1, 7)	0.401
mRS at discharge	2 (1, 4)	2 (1, 4)	0.346
WBC (×10^9^）	7.4 (5.9, 9.5)	7.7 (6.1, 9.7)	**0.024**
NEU (×10^9^）	5.14 (3.85, 7.35)	5.28 (4.05, 7.29)	0.126
LYM (×10^9^）	1.30 (0.91, 1.79)	1.44 (1.04, 1.92)	**<0.001**
MON (×10^9^）	0.46 (0.36, 0.61)	0.46 (0.36, 0.63)	0.572
PLT (×10^9^）	184 (143, 228)	188 (139, 235)	0.680
SII	712 (425, 1,274)	648 (415, 1,219)	0.116
SIRI	1.78 (1.05, 3.44)	1.64 (1.02, 3.24)	0.114
FBG (mmol/L)	6.60 (5.70, 7.93)	11.51 (8.30, 15.4)	**<0.001**
TyG	8.84 (8.44, 9.30)	9.69 (9.18, 10.21)	**<0.001**
HbA1c (%)	5.9 (5.6, 6.2)	7.7 (6.5, 9.6)	**<0.001**
UA (μmol/L)	342.0 (276.0, 419.0)	319.0 (260.0, 396.0)	**0.001**
TC (mmol/L)	4.86 (4.09, 5.62)	5.05 (4.22, 5.92)	**<0.001**
LDL-C (mmol/L)	2.84 (2.28, 3.49)	3.04 (2.42, 3.75)	**<0.001**
HDL-C (mmol/L)	1.32 (1.11, 1.59)	1.28 (1.04, 1.53)	**0.002**
TG (mmol/L)	1.25 (0.90, 1.90)	1.69 (1.15, 2.71)	**<0.001**
RC (mmol/L)	0.52 (0.31, 0.83)	0.57 (0.34, 0.97)	**<0.001**
non-HDL-C (mmol/L)	3.42 (2.77, 4.22)	3.72 (2.94, 4.60)	**<0.001**
AC	2.60 (1.96, 3.35)	2.93 (2.22, 3.79)	**<0.001**
AIP	−0.01 (−0.2, 0.19)	0.14 (−0.07, 0.36)	**<0.001**
LCI	13.00 (6.96, 25.93)	20.60 (10.34, 43.56)	**<0.001**
CRI-I	3.60 (2.96, 4.34)	3.93 (3.22, 4.79)	**<0.001**
CRI-II	2.16 (1.67, 2.78)	2.43 (1.85, 3.06)	**<0.001**
HbA1c/HDL-C	4.48 (3.67, 5.46)	6.27 (4.89, 8.25)	**<0.001**

IS, ischemic stroke; TOAST, Trial of Org 10,172 in Acute Stroke Treatment; CE, Cardioembolism; LAA, large artery atherosclerosis; SAO, small artery occlusion; SOE, stroke of other etiology; SUE, stroke of undetermined etiology; HTN, hypertension; CHD, coronary heart disease; AF, atrial fibrillation; SBP, systolic blood pressure; DBP, diastolic blood pressure; NIHSS, National Institutes of Health Stroke Scale; mRS, modified Rankin Scale; WBC, white blood cell; NEU, neutrophil; LYM, lymphocyte; MON, monocyte; PLT, platelet; SII, systemic inflammation index; SIRI, system inflammation response index; FBG, fasting blood glucose; TyG, triglyceride-glucose index; HbA1c, glycated hemoglobin A1c; UA, uric acid; TC, total cholesterol; LDL-C, low density lipoprotein cholesterol; HDL-C, high density lipoprotein cholesterol; TG, triglyceride; RC, remnant cholesterol; AC, atherogenic coefficient; AIP, atherogenic index of plasma; LCI, lipoprotein combine index; CRI-I, Castelli's risk index I; CRI-II, Castelli's risk index II; bold font indicates statistically significant differences.

**Table 3:** The characteristics of IS patients in the temporal validation set.

Variables	Total (*n* = 406)	IS-only (*n* = 276)	IS-DM (*n* = 130)	*P*-value
Age (years)	71 (62, 79)	71 (64, 79)	68 (59, 77)	**0**.**048**
Gender, *n* (%)				0.592
Female	156 (38)	109 (39)	47 (36)	
Male	250 (62)	167 (61)	83 (64)	
Time of onset (h)	6 (2, 24)	6 (2, 24)	5.5 (2, 24)	0.892
TOAST, *n* (%)				0.097
CE	78 (19)	62 (22)	16 (12)	
LAA	125 (31)	78 (28)	47 (36)	
SAO	185 (46)	122 (44)	63 (48)	
SOE	9 (2)	7 (3)	2 (2)	
SUE	9 (2)	7 (3)	2 (2)	
HTN, *n* (%)				0.135
No	154 (38)	112 (41)	42 (32)	
Yes	252 (62)	164 (59)	88 (68)	
CHD, *n* (%)				0.359
No	372 (92)	250 (91)	122 (94)	
Yes	34 (8)	26 (9)	8 (6)	
AF, *n* (%)				**0**.**032**
No	337 (83)	221 (80)	116 (89)	
Yes	69 (17)	55 (20)	14 (11)	
Antiplatelet therapy, *n* (%)				1
No	397 (98)	270 (98)	127 (98)	
Yes	9 (2)	6 (2)	3 (2)	
Statin therapy, *n* (%)				1
No	397 (98)	270 (98)	127 (98)	
Yes	9 (2)	6 (2)	3 (2)	
Smoking, *n* (%)				0.940
No	293 (72)	200 (72)	93 (72)	
Yes	113 (28)	76 (28)	37 (28)	
Drinking, *n* (%)				0.609
No	320 (79)	220 (80)	100 (77)	
Yes	86 (21)	56 (20)	30 (23)	
SBP	144 ± 25	144 ± 25	143 ± 25	0.604
DBP	83 (73, 94)	84 (73, 96)	82 (73, 93)	0.525
NIHSS at admission	5 (2, 9)	5 (2, 9)	4 (2, 8)	0.382
NIHSS at discharge	4 (1, 8)	4 (1, 8)	4 (1, 8)	0.631
mRS at discharge	3 (1,4)	2 (1,4)	3 (1,4)	0.603
WBC (×10^9^)	7.6 (6.1, 9. 7)	7.5 (6.0, 9.7)	7.9 (6.3, 9.6)	0.263
NEU (×10^9^)	5.30 (4.04, 7.36)	5.23 (4.04, 7.39)	5.43 (4.07, 7.36)	0.517
LYM (×10^9^)	1.35 (0.92, 1.87)	1.28 (0.89, 1.83)	1.50 (1.01, 1.98)	**0**.**047**
MON (×10^9^)	0.47 (0.35, 0.64)	0.46 (0.35, 0.64)	0.48 (0.34, 0.62)	0.669
PLT (×10^9^)	190 (145, 239)	189 (142, 238)	191 (155, 238)	0.758
SII	755 (430, 1,259)	797 (440, 1,257)	669 (410, 1,300)	0.370
SIRI	1.83 (1.10, 3.46)	1.96 (1.17, 3.56)	1.60 (0.96, 3.37)	0.154
FBG (mmol/L)	7.00 (5.80, 9.40)	6.40 (5.51, 7.62)	11.02 (8.12, 14.90)	**<0.001**
TyG	9.04 (8.57, 9.53)	8.79 (8.38, 9.27)	9.54 (9.07, 10.25)	**<0.001**
HbA1c (%)	6.1 (5.7, 6.7)	5.9 (5.6, 6.2)	7.6 (6.3, 9.5)	**<0.001**
UA (μmol/L)	346.5 (280.3, 424.0)	349.5 (281.8, 425.5)	334.5 (266.3, 421.5)	0.459
TC (mmol/L)	4.95 (4.19, 5.86)	4.82 (4.08, 5.71)	5.44 (4.48, 6.08)	**0**.**001**
LDL-C (mmol/L)	3.02 ± 0.97	2.89 ± 0.92	3.29 ± 1.01	**<0.001**
HDL-C (mmol/L)	1.34 (1.11, 1.59)	1.33 (1.10, 1.62)	1.34 (1.14, 1.58)	0.807
TG (mmol/L)	1.40 (0.96, 2.04)	1.29 (0.90, 1.94)	1.67 (1.19, 2.80)	**<0.001**
RC (mmol/L)	0.56 (0.31, 0.91)	0.53 (0.30, 0.88)	0.57 (0.32, 0.99)	0.192
non-HDL-C (mmol/L)	3.62 (2.84, 4.47)	3.44 (2.76, 4.28)	4.07 (3.12, 4.73)	**<0.001**
AC	2.70 (1.98, 3.46)	2.60 (1.89, 3.26)	2.99 (2.32, 3.86)	**<0.001**
AIP	0.04 (−0.18, 0.23)	−0.01 (−0.21, 0.19)	0.09 (−0.08, 0.35)	**<0.001**
LCI	16.45 (7.58, 31.00)	13.13 (6.31, 25.57)	21.80 (11.52, 46.38)	**<0.001**
CRI-I	3.70 (2.98, 4.46)	3.60 (2.89, 4.26)	3.99 (3.32, 4.86)	**<0.001**
CRI-II	2.23 (1.70, 2.82)	2.08 (1.65, 2.67)	2.48 (1.91, 3.08)	**<0.001**
HbA1c/HDL-C	4.77 (3.82, 5.94)	4.42 (3.60, 5.39)	5.74 (4.70, 7.68)	**<0.001**

IS, ischemic stroke; TOAST, Trial of Org 10,172 in Acute Stroke Treatment; CE, Cardioembolism; LAA, large artery atherosclerosis; SAO, small artery occlusion; SOE, stroke of other etiology; SUE, stroke of undetermined etiology; HTN, hypertension; CHD, coronary heart disease; AF, atrial fibrillation; SBP, systolic blood pressure; DBP, diastolic blood pressure; NIHSS, National Institutes of Health Stroke Scale; mRS, modified Rankin Scale; WBC, white blood cell; NEU, neutrophil; LYM, lymphocyte; MON, monocyte; PLT, platelet; SII, systemic inflammation index; SIRI, system inflammation response index; FBG, fasting blood glucose; TyG, triglyceride-glucose index; HbA1c, glycated hemoglobin A1c; UA, uric acid; TC, total cholesterol; LDL-C, low density lipoprotein cholesterol; HDL-C, high density lipoprotein cholesterol; TG, triglyceride; RC, remnant cholesterol; AC, atherogenic coefficient; AIP, atherogenic index of plasma; LCI, lipoprotein combine index; CRI-I, Castelli's risk index I; CRI-II, Castelli's risk index II; bold font indicates statistically significant differences.

### The associations between metabolic parameters and IS-DM comorbidity

**Figure 2:**
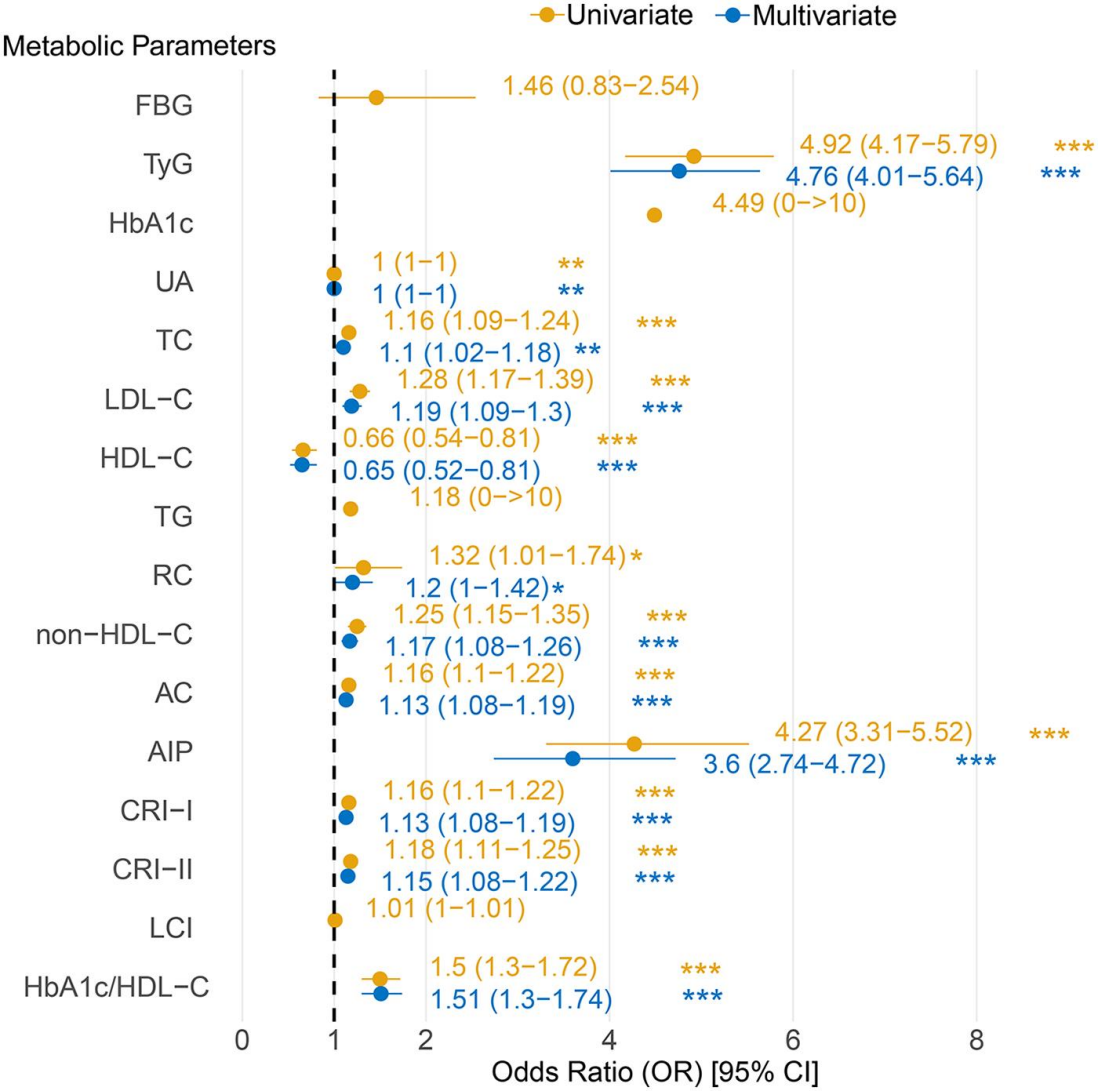
Forest plot showing the associations between 16 metabolic parameters and iS-DM comorbidity. Yellow indicates univariate logistic regression analysis, and blue indicates multivariate logistic regression analysis; *, represents *P* < 0.05; **, represents *P* < 0.01; ***, represents *P* < 0.001.

Univariate analysis revealed no significant associations of FBG, HbA1c, TG, and LCI with IS-DM comorbidity. Following multivariate regression analysis, HDL-C was found to be a protective factor (OR = 0.65, 95% CI: 0.52–0.81, *P* < 0.001), and the remaining 11 metabolic indicators were positively associated with the comorbidity (Figure 2).Notably, TyG (OR = 4.76, 95% CI: 4.01–5.64, *P* < 0.001) and AIP (OR = 3.60, 95% CI: 2.74–4.72, *P* < 0.001) were significantly correlated with elevated IS-DM comorbidity risk (Figure 2).

### RCS analysis

**Figure 3:**
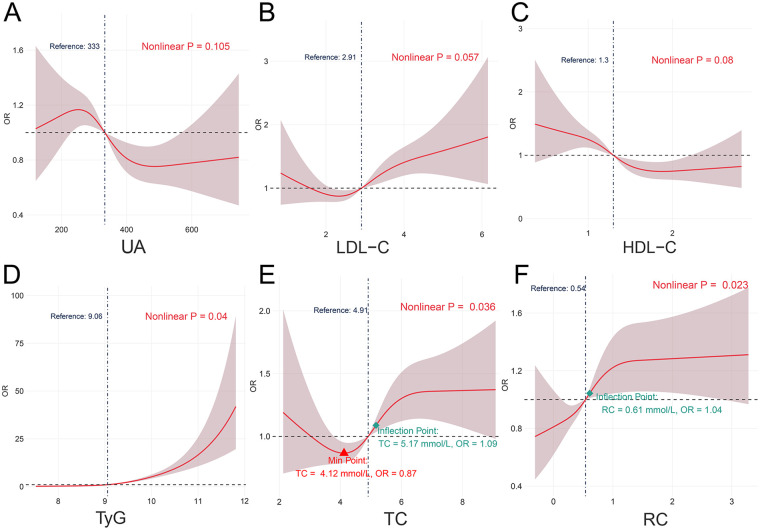
RCS analysis illustrating the non-linear associations between metabolic indicators and IS-DM comorbidity. **(A)** UA, **(B)** LDL-C, **(C)** HDL-C, **(D)** TyG, **(E)** TC, **(F)** RC. The dashed line represents the reference line at an OR of 1. For curves showing significant nonlinear associations, further interpretation should focus on the lowest point and inflection points observed in the fitted curves, which correspond to the optimal concentration with the lowest comorbidity risk and critical cutoffs where risk trends shift markedly, respectively. Shaded areas represent the 95% CIs of fitted OR values. RCS, restricted cubic spline; OR, odds ratio.

**Figure 4:**
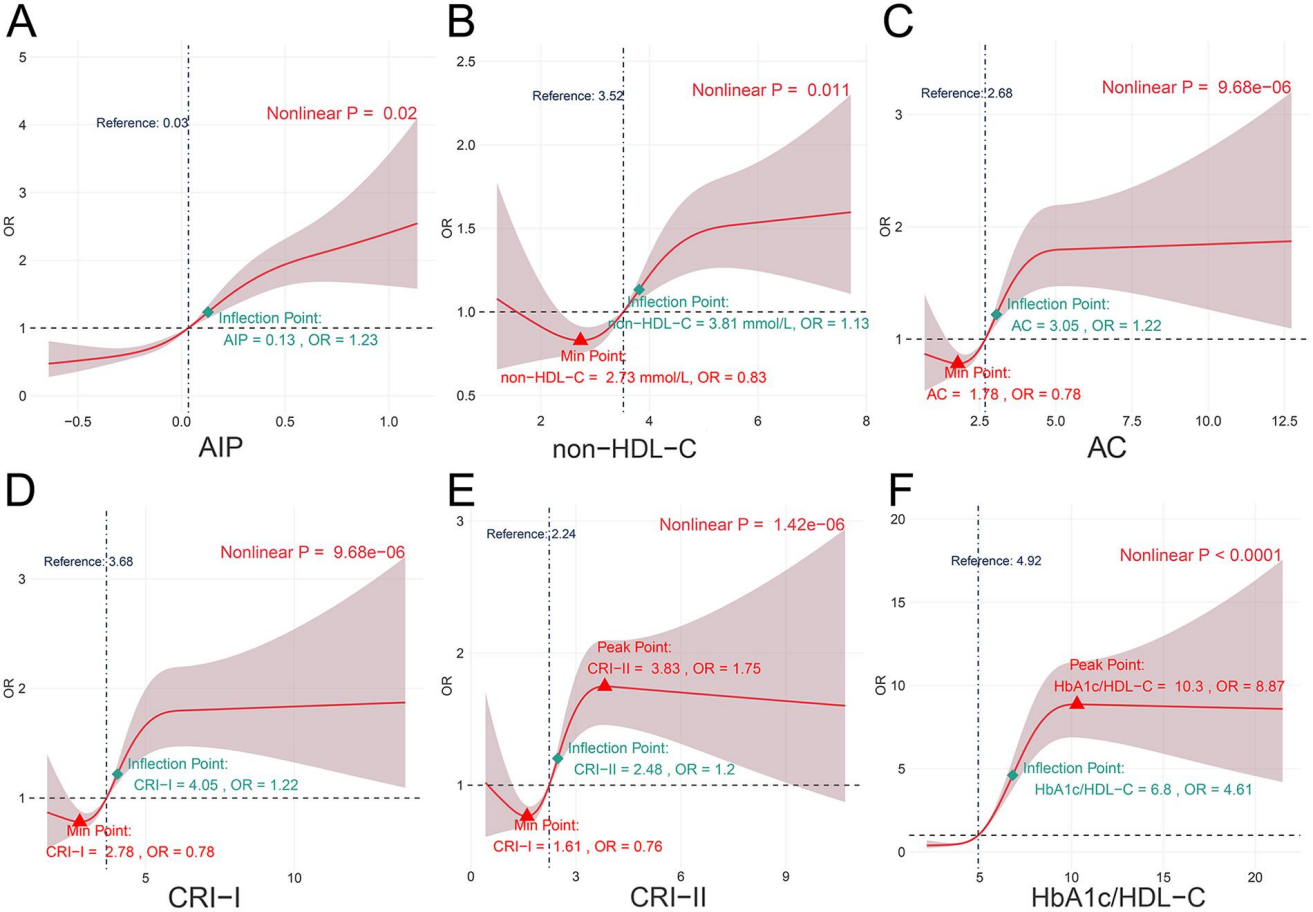
RCS analysis illustrating the non-linear associations between metabolic indicators and IS-DM comorbidity. **(A)** AIP, **(B)** non-HDL-C, **(C)** AC, **(D)** CRI-I, **(E)** CRI-II, **(F)** HbA1c/HDL-C. The dashed line represents the reference line at an OR of 1. For curves with significant nonlinear associations, three key sites including the lowest point, inflection point and peak on the fitted curves deserve further interpretation. Specifically, the lowest point indicates the optimal concentration with the lowest comorbidity risk, inflection points denote critical cutoffs where risk trends shift markedly, and a peak represents the concentration corresponding to maximum disease risk. Shaded areas represent the 95% CIs of fitted OR values. RCS, restricted cubic spline; OR, odds ratio.

RCS analysis was performed to further explore the nonlinear dose-response associations between the above 12 metabolic indicators and IS-DM comorbidity. The results revealed significant nonlinear associations between nine metabolic indicators and comorbidity (*P* for nonlinearity <0.05), except for UA (Figure 3A), LDL-C (Figure 3B) and HDL-C (Figure 3C). TyG exhibited a persistent positive correlation with comorbidity risk, where higher levels were associated with a significantly increased risk (Figure 3D). The risk of comorbidity was lowest at a TC level of 4.12 mmol/L (OR = 0.87) and increased continuously with rising TC levels when ≥5.17 mmol/L (OR = 1.09) (Figure 3E). The risk of comorbidity increased continuously with elevated levels for RC when ≥0.61 mmol/L (OR = 1.04, Figure 3F) and for AIP when ≥0.13 (OR = 1.23, Figure 4A). For non-HDL-C, the risk was lowest at 2.73 mmol/L (OR = 0.83) and increased with rising levels when ≥3.81 mmol/L (OR = 1.13) (Figure 4B). The risk was lowest at 1.78 for AC (OR = 0.78) and increased continuously when ≥3.05 (OR = 1.22) (Figure 4C); lowest at 2.78 for CRI-I (OR = 0.78) and increased continuously when ≥4.05 (OR = 1.22) (Figure 4D); lowest at 1.61 for CRI-II (OR = 0.76), increased continuously when ≥2.48 (OR = 1.2), and peaked at 3.83 (OR = 1.75, Figure 4E). For the HbA1c/HDL-C, the risk increased continuously when ≥6.8 (OR = 4.61) and peaked at 10.3 (OR = 8.87, Figure 4F). These results suggest distinct threshold effects and nonlinear characteristics in the associations between these metabolic indicators and comorbidity risk, with a continuous upward trend in comorbidity risk observed for most indicators after reaching specific inflection points.

### Feature variable selection and development of clinlabomics models

**Figure 5:**
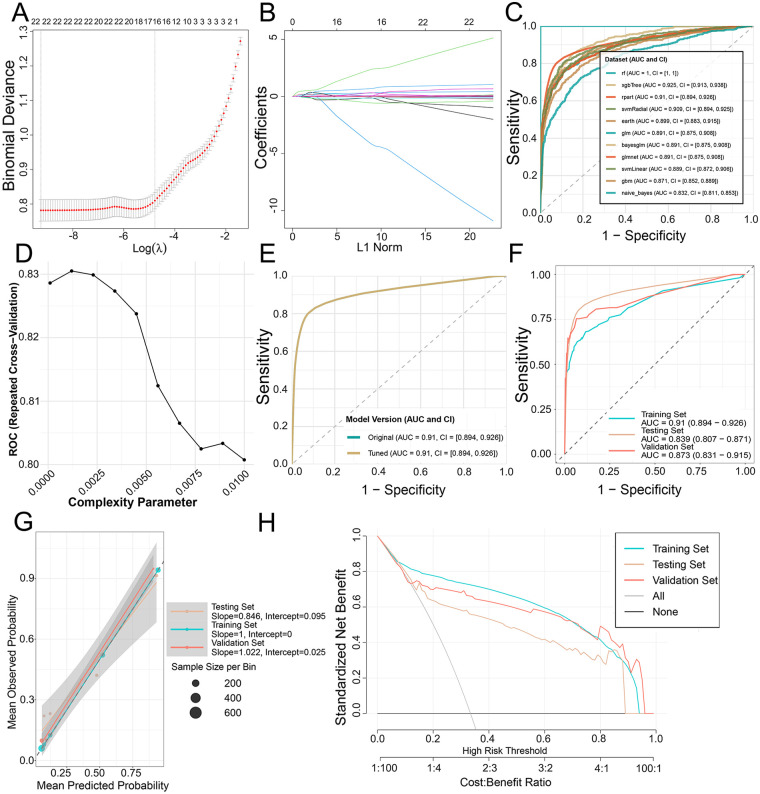
Development of a clinlabomics model for comorbidity prediction. **(A)** Ten-fold cross-validation error curve of LASSO regression. The *x*-axis shows the logarithmic value of the regularization parameter *λ* (Log(*λ*)), and the *y*-axis represents binomial deviance. The red curve indicates model deviance under cross-validation, with the gray shaded area as its confidence interval. The vertical dashed line denotes the optimal *λ* value determined by minimum cross-validation deviance, which balances model complexity and fitting performance, yielding 16 features with non-zero coefficients. **(B)** Coefficient trajectory plot of LASSO regression. The *x*-axis represents the L1 norm, and the *y*-axis shows feature regression coefficients. Curves of different colors illustrate the coefficient variation of each feature with increasing L1 norm. Coefficients of partial features are gradually shrunk to 0, and only 16 non-zero coefficient features significantly associated with comorbidity risk are retained for dimensionality reduction. **(C)** ROC curves of models constructed by 11 machine learning algorithms in the training set. **(D)** Hyperparameter tuning of the optimal rpart model. **(E)** ROC curves, AUC values and 95% CIs of the optimal model in the training set before and after hyperparameter tuning. **(F)** ROC curves of the tuned optimal model in the training, test and validation sets, respectively, for evaluating model discrimination and reliability. **(G)** Calibration curves of the optimal model in the three datasets to assess the consistency between predicted and observed probabilities. **(H)** DCA curves of the optimal model in the three datasets for evaluating the clinical net benefit. LASSO, Least Absolute Shrinkage and Selection Operator; ROC, receiver operating characteristic; DCA, decision curve analysis.

**Figure 6:**
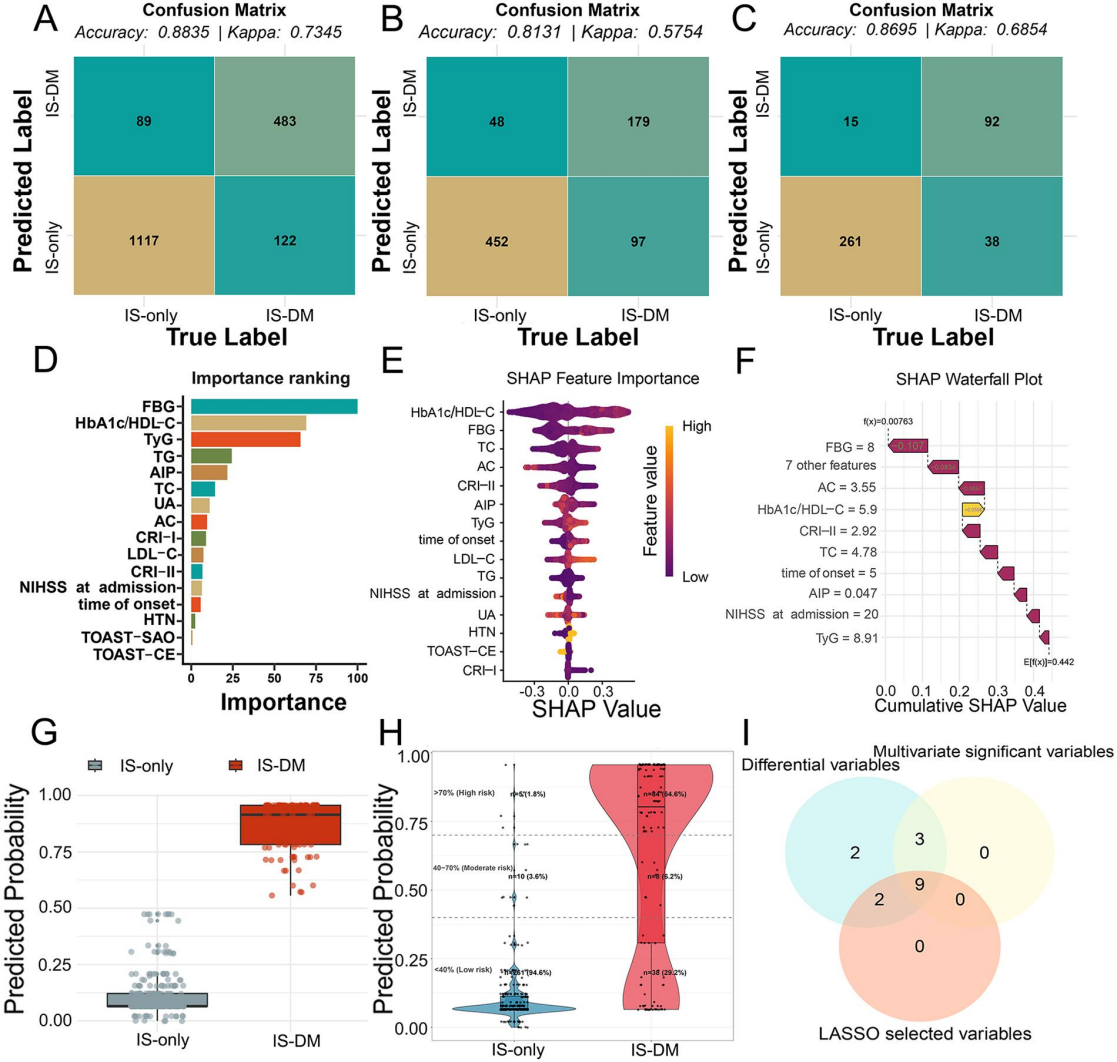
Interpretation of the optimal model and candidate metabolic indicators closely associated with IS-DM comorbidity. **(A–C)** Confusion matrices of the optimal model in the **(A)** training set, **(B)** internal test set and **(C)** temporal validation set, showing the classification accuracy and Kappa coefficient of each cohort for evaluating classification accuracy and consistency. **(D)** Feature importance ranking for identifying key feature variables in the model. **(E)** SHAP beeswarm plot illustrating the overall direction and magnitude of each feature's predictive effect on model output. The *x*-axis represents SHAP values (reflecting the contribution of each feature to model output), and the *y*-axis lists feature names; colors indicate feature levels (yellow for high values, purple for low values), intuitively showing the contribution ranking of features to comorbidity risk assessment, with HbA1c/HDL-C as the core feature. **(F)** SHAP waterfall plot for a single sample. Based on the average predicted risk of all samples (E[f**(x)**] = 0.442), the final predicted risk of this sample (f**(x)** = 0.00763) is obtained by summing the SHAP values of individual features; red indicates risk-increasing effects and yellow indicates risk-reducing effects, clearly quantifying the direction and magnitude of each feature's impact on individual prediction. **(G)** Boxplot of patient classification into comorbidity and non-comorbidity groups in the validation cohort by the model after label removal. **(H)** Violin plot showing the distribution of predicted probabilities from the optimal Clinlabomics model across different risk stratifications and actual groups. Low, intermediate and high risk were defined as predicted probability <40%, 40%–70% and ≥70%, respectively. The plot shows the number and proportion of IS-only and IS-DM comorbidity cases in each stratum, reflecting the model's risk stratification performance for IS-only (94.6%) and comorbidity (64.6%) populations. **(I)** Venn diagram showing the overlap of three sets of metabolic variables: differential metabolic variables in the training set (light blue), variables significantly associated in multivariate logistic regression (light yellow), and modeling variables selected by LASSO regression (light orange). Numbers represent the count of variables in each set and their intersections. The shared intersection contains 9 variables representing core metabolic features significantly associated with IS-DM comorbidity risk.

During model construction, LASSO regression was applied to select feature variables for differential clinical and metabolic features in the training set, screening 16 important features (Figures 5A,B). After comprehensive evaluation of ACC, F1-score, AUC, sensitivity and specificity, the recursive partitioning and regression trees (rpart) algorithm was determined as the optimal one among 11 Clinlabomics models established by ML algorithms, with an ACC of 0.885, F1-score of 0.818, AUC of 0.910, sensitivity of 78%, and specificity of 94% in the training set (Figure 5C). Hyperparameter tuning identified the optimal cp value as 0.00096, with no obvious improvement in AUC (Figures 5D,E). ROC curve presented an AUC of 0.910 (95% CI: 0.894–0.926), 0.839 (95% CI: 0.807–0.871), and 0.873 (95% CI: 0.831–0.915) in the training, test, and validation sets, respectively (Figure 5F). Calibration curves revealed high consistency between predicted and observed risks across the three cohorts, with slopes close to 1 (Figure 5G). The Brier scores of the model in the training, test and validation sets were 0.093, 0.143 and 0.103, respectively. A Brier score approaching 0 indicates minimal prediction bias, high predictive accuracy and satisfactory calibration, further demonstrating good model performance. DCA showed that within a broad range of risk thresholds, the standardized net benefit of the model was consistently higher than the extreme strategies of “no intervention” and “full-population intervention”, with consistent trends across cohorts, supporting the clinical value of the model (Figure 5H). Confusion matrix and Kappa tests confirmed stable performance and good consistency (Figures 6A–C). Feature importance and SHAP analysis recognized FBG, HbA1c/HDL-C, and TyG as key metabolic indicators, with HbA1c/HDL-C as the core feature (Figures 6D,E). SHAP waterfall plots further quantified the direction and magnitude of risk contribution for each feature in individual samples, improving the interpretability of personalized model evaluation (Figure 6F). In the temporal validation set, the model identified 89 patients with comorbidity (risk threshold >70%) and 299 patients without comorbidity (risk threshold <40%) (Figure 6G). The model demonstrated 94.6% accuracy (*n* = 261) in identifying IS-only patients and 64.6% (*n* = 84) in detecting high-risk IS-DM comorbidity patients, underscoring its advantage in identifying IS-only individuals while retaining reliable comorbidity recognition ability (Figure 6H). Venn diagram identified 9 candidate metabolic features significantly related to comorbidity, including TyG, UA, TC, LDL-C, AIP, AC, CRI-I, CRI-II, and HbA1c/HDL-C (Figure 6I). After excluding HbA1c (excluded by LASSO analysis), FBG and the TyG index, we reconstructed the model using the remaining 14 variables. The rpart algorithm yielded stable predictive performance (Supplementary Figure S1A). The reconstructed model presented an optimal cp hyperparameter of 0.00015 (Supplementary Figure S1B), and the ROC curves were nearly identical between the hyperparameter-optimized and unoptimized models (Supplementary Figure S1C). The model achieved an AUC of 0.916 (95% CI: 0.902–0.930) with 76% sensitivity and 94% specificity in the training set. The corresponding AUC values were 0.806 (95% CI: 0.772–0.839) and 0.862 (95% CI: 0.822–0.903) in the test and temporal validation sets, respectively (Supplementary Figure S1D). Calibration and decision curve analyses (Supplementary Figures S1E,F) demonstrated optimal probability calibration and favorable clinical utility. The Brier scores of 0.097, 0.172 and 0.130 across three cohorts reflected low prediction errors, further supporting reliable model performance. Variable importance and SHAP summary plots verified that the HbA1c/HDL-C ratio remained the dominant predictive variable (Supplementary Figures S1G,H). In the temporal validation cohort, the model attained an accuracy of 89.5% (*n* = 247) for IS-only patients and 53.1% (*n* = 69) for patients with IS-DM comorbidity (Supplementary Figure S1I). The between-group performance discrepancy was an expected change following the removal of glycaemic markers. Notably, the model retained adequate discriminative capacity without HbA1c, FBG, and TyG, proving its predictive performance was not driven by diagnostic indicators for diabetes. Collectively, these results rule out the risk of circular reasoning in this study.

## Discussion

Through integrative analysis, this study identified nine candidate metabolic indicators significantly associated with IS-DM comorbidity, including TyG, UA, TC, LDL-C, AIP, AC, CRI-I, CRI-II, and HbA1c/HDL-C. We further established Clinlabomics models for comorbidity using 11 ML algorithms, with the rpart algorithm exhibiting optimal performance. It can effectively identify patients at high risk of IS-DM comorbidity and has promising application prospects in clinical practice.

Clinlabomics, first proposed by Wang et al. in 2022, integrates moderate-dimensional laboratory indicators and clinical characteristics with ML algorithms to construct models for disease warning or prognostic prediction (11). For instance, Nie et al. used peripheral blood data and ML algorithms to develop ICI therapeutic response prediction Clinlabomics models for advanced gastric cancer, with the XGBoost algorithm achieving the best performance (training set: AUC = 0.863; validation set: AUC ranging from 0.790 to 0.842) (12). Luo et al. (15) developed a Clinlabomics-based nomogram for prognostic evaluation in Chinese small cell lung cancer patients, which showed good accuracy for 2- and 3-year OS (training set: AUC = 0.74 and 0.74; validation set: AUC = 0.64 and 0.74, *P* < 0.001). We previously used unsupervised clustering to identify AIS phenotypes and adopted SVM algorithm to develop phenotype-specific Clinlabomics models, with AUC and accuracy both > 0.90 (14). Cai et al. (29) constructed Clinlabomics models for large artery atherosclerotic IS using LR, SVM and RF, and the RF-based model exhibited excellent performance (AUC = 0.955) with promising clinical applicability.

In the present study, the IS-DM comorbidity model established using the optimal rpart algorithm achieved AUC values above 0.80 across all three datasets. It maintained reliable predictive performance among participants recruited at different time points in this single center. Clinlabomics allows disease risk assessment by integrating clinical and routine laboratory data through ML algorithms (11). Our team previously established Clinlabomics models for different AIS phenotypes (14) and IS-HUA comorbidity (16), enabling early identification of high-risk individuals. Consistent with prior results, the rpart algorithm remained superior in identification of IS-DM comorbidity, confirming its stability and universality, and providing reliable methodological support for this study.

SHAP analysis revealed that HbA1c/HDL-C played a critical role in the present comorbidity model. Likewise, our previous study, also based on SHAP algorithm, identified that uric acid (UA) served as a core feature for identifying the comorbidity of IS and HUA (16). In addition, Huang et al. applied SHAP methodology to interpret key model features, which effectively unpacked the “black box” nature of ML models, enabled intuitive and visual quantification of each feature's contribution, and substantially improved model interpretability and transparency (30). Of note, the HbA1c/HDL-C ratio, a novel composite marker for glycemic homeostasis and dyslipidemia, has been rarely studied, representing a key innovation of this study. Notably, in this study, HbA1c/HDL-C was significantly associated with an increased risk of IS-DM comorbidity (OR = 1.51, 95% CI: 1.30–1.74), and the comorbidity risk peaked at values above 10.3 (OR = 8.87; *P* for nonlinearity <0.001). Besides, elevated HbA1c is significantly associated with an increased risk of IS in diabetic patients (31). HbA1c reflects average blood glucose over 2–3 months and serves as a key indicator in DM management. HDL-C mediates reverse cholesterol transport and endothelial protection, and its reduction is a major risk factor for atherosclerosis. An increased HbA1c/HDL-C ratio indicates concurrent poor glycemic control and decreased protective lipoproteins, aggravating vascular injury through metabolic disorders and exerting a synergistic effect in IS-DM comorbidity. As an integrated metabolic biomarker, this ratio provides comprehensive evidence for risk assessment and overcomes the limitation of single indicators in evaluating dual glucolipid abnormalities. Notably, to avoid circular reasoning, we excluded FBG, TyG, and HbA1c (which had been eliminated during LASSO analysis), and conducted sensitivity analysis with the remaining 14 variables. The reconstructed model performed steadily, and the HbA1c/HDL-C ratio remained the dominant predictor. The slight difference in classification performance across subgroups in temporal validation was a normal change after excluding glycaemic parameters. The retained discriminative capacity fully rules out the presence of circular reasoning.

Subsequently, by integrating differential metabolic parameters, multivariate logistic regression and LASSO analysis, we identified nine candidate metabolic signatures linked to comorbidity via Venn diagram (TyG, UA, TC, LDL-C, AIP, AC, CRI-I, CRI-II, and HbA1c/HDL-C). These nine metabolic indicators may serve as promising biomarkers for comorbidity assessment and provide a reliable foundation for future mechanistic and clinical translational studies. Derived entirely from routine clinical lipid profiles with no extra assays required, these indicators possess excellent clinical accessibility and translational potential. TyG, a classic metabolic marker of insulin resistance (IR), inhibits insulin signaling in intimal cells, inducing endothelial dysfunction, chronic inflammation and aberrant oxidative stress responses. It promotes foam cell formation, vulnerable plaque development and plaque necrosis, ultimately driving the formation and rupture of atherosclerotic plaques (32, 33). As a core pathological process in T2DM, TyG also acts as an important independent risk factor for IS. A UK Biobank study revealed that elevated TyG-related indices were associated with increased IS risk, and each 1-unit increment in TyG significantly raised stroke risk in diabetic patients (34). Guo et al. reported that higher TyG levels correlated with elevated prevalence of HTN and DM, abnormal lipid and metabolic profiles, and altered carotid plaque features (35). Besides, TyG was significantly associated with early neurological deterioration (END) in IS patients (OR = 2.22, 95% CI: 1.51–3.27) (36). A meta-analysis demonstrated that high TyG was linked to elevated risks of T2DM (RR = 3.53, 95% CI: 2.74–4.54) and IS (OR = 1.37, 95% CI: 1.22–1.54) (37). In this study, TyG was identified as an important indicator of IS-DM comorbidity and significantly associated with an increased risk of comorbidity (OR = 4.76, 95% CI: 4.01–5.64, *P* < 0.001; *P* for nonlinearity = 0.04). Thus, TyG can serve as a reliable metabolic biomarker for evaluating IS-DM.

Accumulating evidence shows that elevated serum UA impairs insulin sensitivity and *β*-cell function via oxidative stress, inflammation and urate transporter dysfunction. Insulin resistance in turn reduces renal urate excretion, forming a vicious cycle (38). Mendelian randomization confirmed a causal link between UA and IS (OR = 1.23, 95% CI: 1.13–1.34, *P* = 6.39 × 10^−9^), with stronger effects on large-vessel and small-vessel stroke. T2DM mediated these associations, with mediating proportions of 13.85% and 13.57% respectively (39). A prior study reported that a 0–50 μmol/L (OR = 0.46, 95% CI: 0.28–0.78, *P* = 0.004) or 50–100 μmol/L (OR = 0.40, 95% CI: 0.21–0.77, *P* = 0.006) decline in UA within three months after stroke onset correlated with lower risks of adverse neurological outcomes in IS-DM population (40). Contrary to theoretical expectations, UA levels were lower in the IS-DM group than in IS-only group in this study. After confounder adjustment, UA remained a risk factor for IS-DM comorbidity (OR = 1.00, 95% CI: 1.00–1.00, *P* < 0.05). This discrepancy is mainly attributed to early diabetic nephropathy, administration of urate-lowering hypoglycemic drugs, and the limitation of cross-sectional design which fails to reflect pre-onset baseline UA. Prospective cohort studies are needed to further clarify the relationship between UA and IS-DM comorbidity.

The CRI-I, CRI-II, and AIP are positively associated with prediabetes and T2DM in IS patients, with AIP showing the strongest correlation (41). Among 222 IS patients undergoing mechanical thrombectomy, Boncuk Ulaş S et al. found that higher AIP levels in diabetic patients and a positive correlation of AIP with diabetes duration and prior stroke (42), supporting its role as an integrated biomarker for glucose-lipid-related cerebrovascular damage. Likewise, we demonstrated that AIP was positively associated with comorbidity risk (OR = 3.60, 95% CI: 2.74–4.72, *P* < 0.001; *P* for nonlinearity = 0.02). Our previous work revealed higher levels of TC, LDL-C, AIP, AC, CRI-I, and CRI-II in distinct AIS phenotypes, indicating obvious lipid disturbances (14). Elevated CRI-I, CRI-II, and AC increase carotid atherosclerosis risk in IS patients (43), while AIP, CRI-I, CRI-II, and AC can predict intracranial and extracranial atherosclerotic stenosis, especially AIP (26). AC, also known as the non-HDL-C/HDL-C ratio (NHHR), is associated with cardiovascular mortality in diabetes (HR = 1.39, 95% CI: 1.11–1.73) (44). Similarly, in this study, AC was positively associated with IS-DM comorbidity (OR = 1.13, 95% CI: 1.08–1.19), and the risk of comorbidity was significantly increased when AC exceeded 1.78 (*P* for nonlinearity < 0.001). In summary, these non-traditional lipid markers reflect multifaceted atherogenic lipid abnormalities. By synergistically worsening endothelial injury and promoting atherosclerotic plaque progression, they act as critical pathological links between DM and IS.

**Figure 7:**
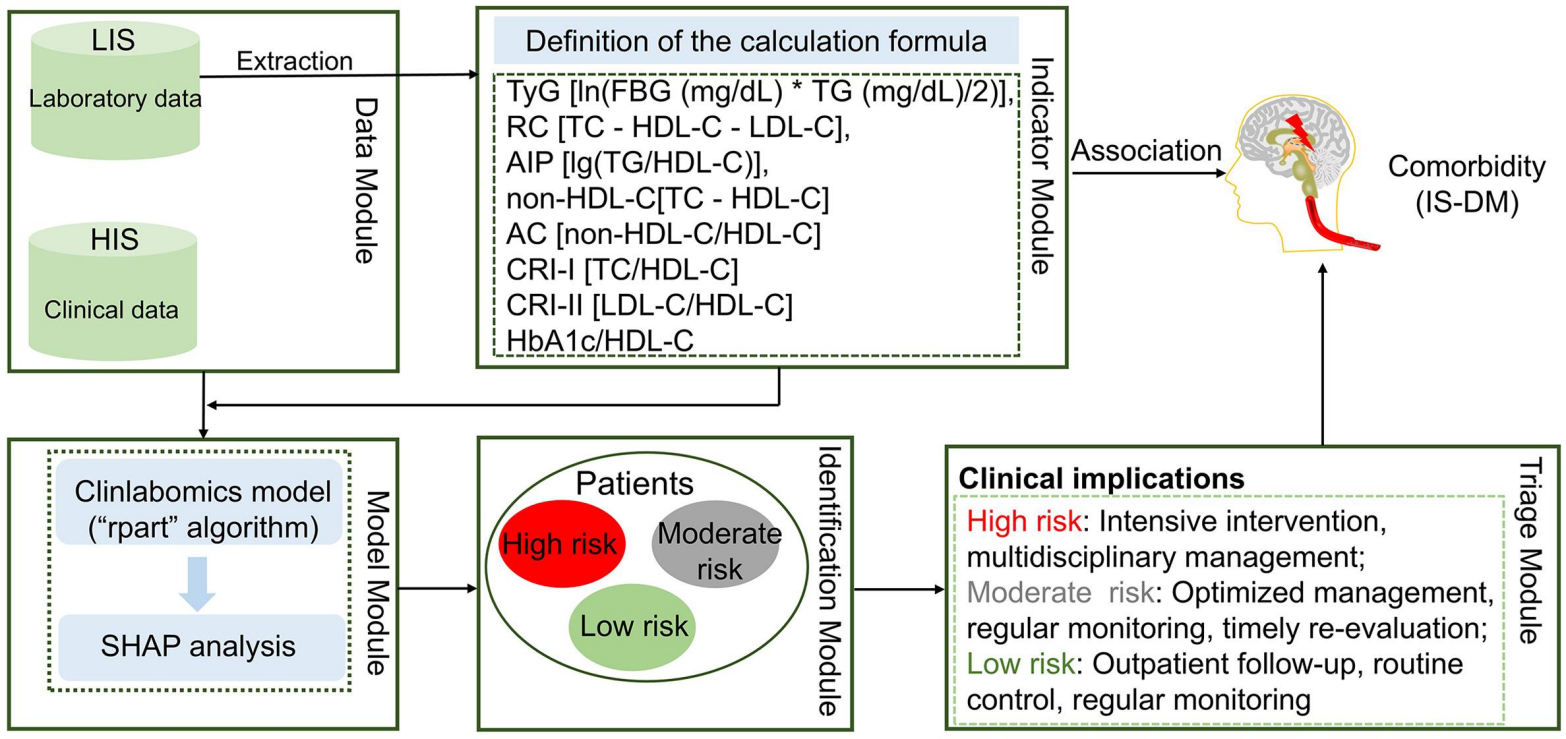
Schematic of the clinlabomics-based screening and identification model for IS-DM comorbidity and clinical triage workflow. The flowchart illustrates the full workflow: (1) Data Module: Extraction of laboratory and clinical data from LIS and HIS, respectively; (2) Indicator Module: Standardized calculation of derived metabolic indicators; (3) Model Module: Development of the rpart-based Clinlabomics model with SHAP interpretation; (4) Identification Module: Identification of patients by risk probability (high, moderate, low); (5) Triage Module: Implementation of differentiated triage management strategies for different risk population, supporting the clinical identification and hierarchical management of IS-DM comorbidity. LIS, laboratory information system; HIS, hospital information system; SHAP, SHapley Additive exPlanations.

All factors in the optimal Clinlabomics model, including TyG and AIP, are calculable directly from routine clinical biochemical tests. They add no extra testing burden or medical costs to patients, and the relevant laboratory data can be directly obtained in clinical practice and fed into the models for rapid identification of IS-DM comorbidity. Standard diagnostic criteria for diabetes merely confirm disease presence and are incapable of rapid risk stratification for stroke patients in emergency and inpatient care. Established on metabolic parameters, the Clinlabomics model effectively identifies patients at high risk of undiagnosed IS-DM comorbidity and supports individualized treatment. This distinct advantage remedies the shortcomings of current clinical assessment frameworks. This enables clinicians to perform efficient risk assessments, providing a practical tool for the standardized management of comorbid patients. To facilitate the integration of the Clinlabomics model into routine clinical workflows, we constructed a schematic flowchart illustrating the entire procedure from data extraction and model development to the identification of high- and low-risk patients and subsequent clinical triage (Figure 7).

However, this study has several key limitations. First, as a retrospective single-center study, our findings require validation in multi-center, large-sample prospective cohorts. Second, we only identified correlations between these metabolic indicators and IS-DM comorbidity, without clarifying the underlying molecular mechanisms and causal pathways; further basic research is warranted. Third, only routine clinical and laboratory data were included, without integrating imaging, genetic or lifestyle data, leaving room for improved performance. Last but not least, acute stroke-related physiological stress may affect patients' laboratory test values at admission, which may introduce potential bias into model construction and interpretation.

## Conclusions

This study identified TyG, UA, TC, LDL-C, AIP, AC, CRI-I, CRI-II, and HbA1c/HDL-C as candidate metabolic indicators for IS-DM comorbidity, especially HbA1c/HDL-C. The Clinlabomics comorbidity model constructed by the rpart algorithm shows favorable performance. These findings offer clinically applicable metabolic markers and a reliable interpretable model for the rapid recognition of IS-DM comorbidity. Nevertheless, large-sample, multi-center and prospective cohort studies are still required to validate our findings.

## Data Availability

The original contributions presented in the study are included in the article/Supplementary Material, further inquiries can be directed to the corresponding author/s.
