# Supplementary material for: Identification of key metabolic indicators associated with the comorbidity of ischemic stroke and diabetes mellitus using an optimal interpretable clinlabomics model
Source: Front Cardiovasc Med. 2026 Jun 24;13:1874711. doi: 10.3389/fcvm.2026.1874711 (PMC13341514; doi:10.3389/fcvm.2026.1874711)
Supplement: Supplementary file 1 [file Table1.docx]

**Supplementary Table 1 Missing data overview and comparisons between complete cases and imputed datasets.**

| Variables | Missing (%) | Without missing | After impute | *P*-value |
| --- | --- | --- | --- | --- |
| WBC | 50 (1.9) | 7.5 (6.0, 9.5) | 7.5 (6.0, 9.5) | 0.643 |
| NEU | 50 (1.9) | 5.25 (3.94, 7.30) | 5.25 (3.94, 7.28) | 0.958 |
| LYM | 50 (1.9) | 1.36 (0.97, 1.84) | 1.34 (0.94, 1.83) | 0.305 |
| MON | 50 (1.9) | 0.47 (0.36, 0.62) | 0.47 (0.36, 0.62) | 0.935 |
| PLT | 50 (1.9) | 188 (145, 233) | 187 (142, 232) | 0.449 |
| FBG | 159 (6.1) | 7.24 (6.00, 9.85) | 7.40 (6.04, 10.3) | 0.083 |
| HbA1c | 299 (11.6) | 6.2 (5.7, 7.1) | 6.2 (5.8, 6.9) | 0.889 |
| UA | 36 (1.3) | 333 (270, 407) | 333 (270, 407) | 0.958 |
| TC | 182 (7.0) | 4.90 (4.07, 5.71) | 4.91 (4.10, 5.71) | 0.690 |
| LDL-C | 182 (7.0) | 2.91 (2.32, 3.51) | 2.91 (2.31, 3.53) | 0.989 |
| HDL-C | 182 (7.0) | 1.30 (1.10, 1.55) | 1.30 (1.09, 1.56) | 0.922 |
| TG | 182 (7.0) | 1.34 (0.96, 1.98) | 1.38 (0.97, 2.06) | 0.126 |

WBC, white blood cell; NEU, neutrophil; LYM, lymphocyte; MON, monocyte; PLT, platelet; FBG, fasting blood glucose; HbA1c, glycated hemoglobin A1c; UA, uric acid; TC, total cholesterol; LDL-C, low density lipoprotein cholesterol; HDL-C, high density lipoprotein cholesterol; TG, triglyceride.
