# Supplementary material for: Identification of key metabolic indicators associated with the comorbidity of ischemic stroke and diabetes mellitus using an optimal interpretable clinlabomics model
Source: Front Cardiovasc Med. 2026 Jun 24;13:1874711. doi: 10.3389/fcvm.2026.1874711 (PMC13341514; doi:10.3389/fcvm.2026.1874711)
Supplement: Supplementary file 2 [file Table2.docx]

**Supplementary Table 2 The basic characteristics of IS patients in the test set.**

| Variables | IS-only (n = 500) | IS-DM (n = 276) | *P*-value |
| --- | --- | --- | --- |
| Age (years) | 73 (66, 79) | 71 (64, 79) | 0.121 |
| Gender, n (%) |  |  | **0.025** |
| Female | 198 (40) | 133 (48) |  |
| Male | 302 (60) | 143 (52) |  |
| Time of onset (h) | 5 (2, 24) | 6 (2, 24) | 0.962 |
| TOAST, n (%) |  |  | **< 0.001** |
| CE | 113 (23) | 31 (11) |  |
| LAA | 147 (29) | 75 (27) |  |
| SAO | 218 (44) | 160 (58) |  |
| SOE | 16 (3) | 4 (2) |  |
| SUE | 6 (1) | 6 (2) |  |
| HTN, n (%) |  |  | **0.002** |
| No | 228 (46) | 93 (34) |  |
| Yes | 272 (54) | 183 (66) |  |
| CHD, n (%) |  |  | 0.994 |
| No | 458 (92) | 252 (91) |  |
| Yes | 42 (8) | 24 (9) |  |
| AF, n (%) |  |  | **< 0.001** |
| No | 395 (79) | 251 (91) |  |
| Yes | 105 (21) | 25 (9) |  |
| Antiplatelet therapy, n (%) |  |  | 1 |
| No | 491 (98) | 272 (99) |  |
| Yes | 9 (2) | 4 (1) |  |
| Statin therapy, n (%) |  |  | 1 |
| No | 492 (98) | 272 (99) |  |
| Yes | 8 (2) | 4 (1) |  |
| Smoking, n (%) |  |  | 0.162 |
| No | 350 (70) | 207 (75) |  |
| Yes | 150 (30) | 69 (25) |  |
| Drinking, n (%) |  |  | 0.676 |
| No | 400 (80) | 225 (82) |  |
| Yes | 100 (20) | 51 (18) |  |
| SBP | 146 (128, 162) | 148 (131, 167) | 0.165 |
| DBP | 85 (75, 96) | 85 (75, 94) | 0.742 |
| NIHSS at admission | 5 (2, 10) | 4 (2, 8) | 0.057 |
| NIHSS at discharge | 3 (1, 7) | 3 (2, 8) | 0.713 |
| mRS at discharge | 2 (1, 4) | 2 (1, 4) | 0.4888 |
| WBC (×10^9） | 7.4 (5.8, 9.4) | 7.8 (6.4, 9.4) | **0.047** |
| NEU (×10^9） | 5.20 (3.95, 7.09) | 5.54 (4.22, 7.20) | 0.161 |
| LYM (×10^9） | 1.29 (0.92, 1.80) | 1.43 (1.05, 1.93) | **0.007** |
| MON (×10^9） | 0.47 (0.36, 0.63) | 0.47 (0.35, 0.63) | 0.848 |
| PLT (×10^9） | 184 (141, 238) | 195 (152, 233) | 0.296 |
| SII | 730 (431, 1,257) | 675 (453, 1,159) | 0.624 |
| SIRI | 1.81 (1.16, 3.34) | 1.69 (1.02, 3.17) | 0.21 |
| FBG (mmol/L) | 6.53 (5.58, 8.00) | 11.00 (7.94, 15.57) | **< 0.001** |
| TyG | 8.77 (8.39, 9.18) | 9.64 (9.11, 10.20) | **< 0.001** |
| HbA1c (%) | 5.9 (5.6, 6.2) | 7.8 (6.5, 9.5) | **< 0.001** |
| UA (μmol/L) | 334.0 (272.0, 404.0) | 322.5 (263.0, 397.0) | 0.167 |
| TC (mmol/L) | 4.72 (3.92, 5.50) | 5.11 (4.42, 5.92) | **< 0.001** |
| LDL-C (mmol/L) | 2.74 (2.19, 3.34) | 3.17 (2.43, 3.69) | **< 0.001** |
| HDL-C (mmol/L) | 1.35 (1.14, 1.62) | 1.24 (1.04, 1.49) | **< 0.001** |
| TG (mmol/L) | 1.20 (0.86, 1.71) | 1.70 (1.28, 2.47) | **< 0.001** |
| RC (mmol/L) | 0.50 (0.30, 0.79) | 0.61 (0.39, 0.97) | **< 0.001** |
| non-HDL-C (mmol/L) | 3.27 (2.62, 4.01) | 3.84 (3.13, 4.51) | **< 0.001** |
| AC | 2.39 (1.82, 3.19) | 2.99 (2.38, 3.96) | **< 0.001** |
| AIP | -0.04 (-0.23, 0.14) | 0.12 (-0.03, 0.33) | **< 0.001** |
| LCI | 11.11 (6.12, 21.6) | 21.16 (11.65, 41.92) | **< 0.001** |
| CRI-I | 3.39 (2.82, 4.19) | 3.99 (3.38, 4.96) | **< 0.001** |
| CRI-II | 1.98 (1.52, 2.67) | 2.47 (1.92, 3.08) | **< 0.001** |
| HbA1c/HDL-C | 4.32 (3.61, 5.25) | 6.60 (5.08, 8.38) | **< 0.001** |

IS, ischemic stroke; TOAST, Trial of Org 10172 in Acute Stroke Treatment; CE, Cardioembolism; LAA, large artery atherosclerosis; SAO, small artery occlusion; SOE, stroke of other etiology; SUE, stroke of undetermined etiology; HTN, hypertension; CHD, coronary heart disease; AF, atrial fibrillation; SBP, systolic blood pressure; DBP, diastolic blood pressure; NIHSS, National Institutes of Health Stroke Scale; mRS, modified Rankin Scale; WBC, white blood cell; NEU, neutrophil; LYM, lymphocyte; MON, monocyte; PLT, platelet; SII, systemic inflammation index; SIRI, system inflammation response index; FBG, fasting blood glucose; TyG, triglyceride-glucose index; HbA1c, glycated hemoglobin A1c; UA, uric acid; TC, total cholesterol; LDL-C, low density lipoprotein cholesterol; HDL-C, high density lipoprotein cholesterol; TG, triglyceride; RC, remnant cholesterol; AC, atherogenic coefficient; AIP, atherogenic index of plasma; LCI, lipoprotein combine index; CRI-I, Castelli’s risk index I; CRI-II, Castelli’s risk index II; bold font indicates statistically significant differences.
