# Supplementary figures and images for: Identification of key metabolic indicators associated with the comorbidity of ischemic stroke and diabetes mellitus using an optimal interpretable clinlabomics model
Source: Front Cardiovasc Med. 2026 Jun 24;13:1874711. doi: 10.3389/fcvm.2026.1874711 (PMC13341514; doi:10.3389/fcvm.2026.1874711)

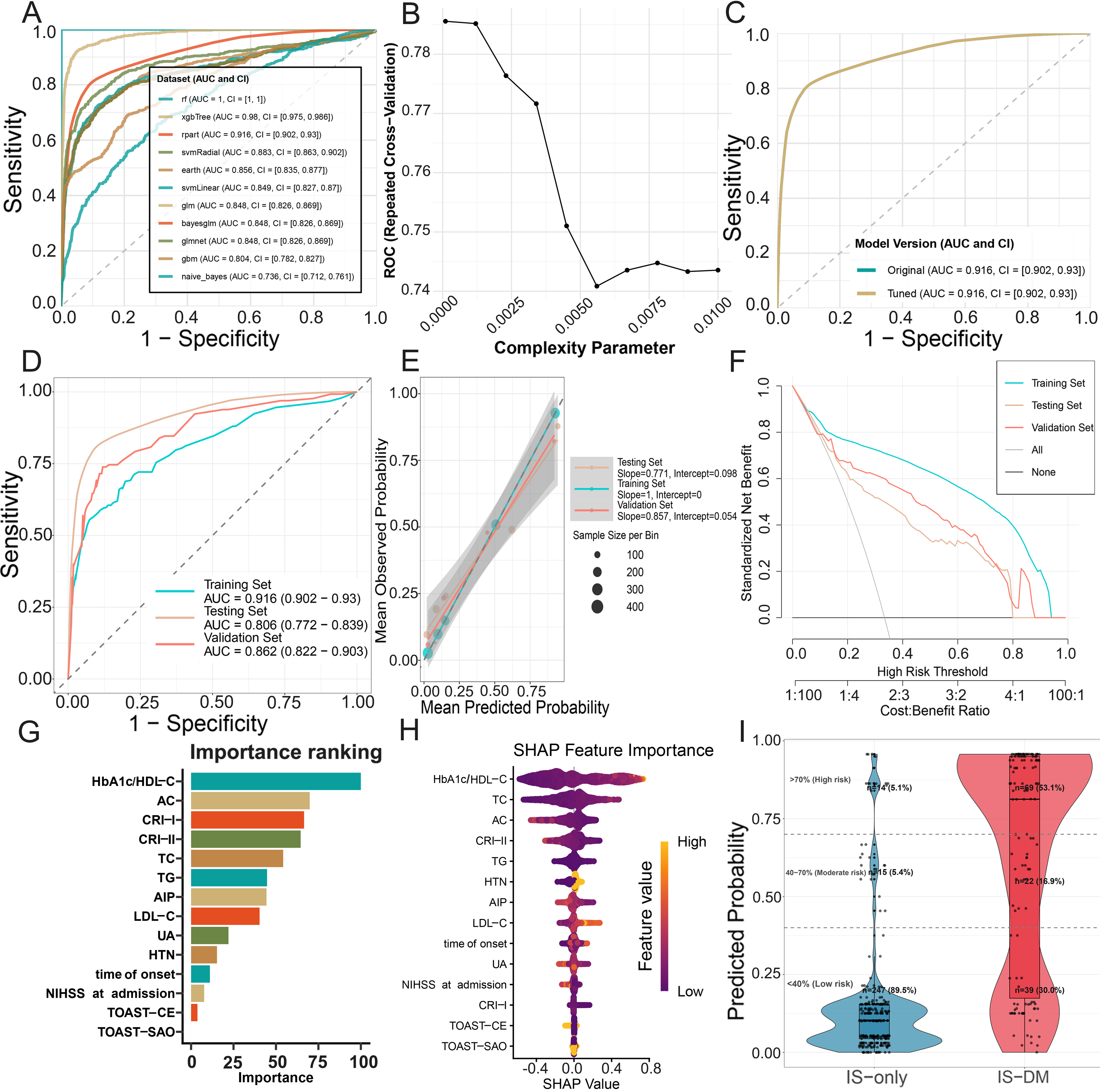

Supplement: Supplementary file 3 [file Image1.tif]
